# Differentiation of human induced pluripotent stem cells into hypothalamic vasopressin neurons with minimal exogenous signals and partial conversion to the naive state

**DOI:** 10.1038/s41598-022-22405-8

**Published:** 2022-10-17

**Authors:** Hajime Ozaki, Hidetaka Suga, Mayu Sakakibara, Mika Soen, Natsuki Miyake, Tsutomu Miwata, Shiori Taga, Takashi Nagai, Mayuko Kano, Kazuki Mitsumoto, Takashi Miyata, Tomoko Kobayashi, Mariko Sugiyama, Takeshi Onoue, Hiroshi Takagi, Daisuke Hagiwara, Shintaro Iwama, Ryoichi Banno, Genzo Iguchi, Yutaka Takahashi, Keiko Muguruma, Haruhisa Inoue, Hiroshi Arima

**Affiliations:** 1 Department of Endocrinology and Diabetes, Nagoya University Graduate School of Medicine, 65 Tsurumai-cho, Showa-ku, Nagoya, 466-8550 Japan; 2 Department of Obstetrics and Gynecology, Nagoya University Graduate School of Medicine, 65 Tsurumai-cho, Showa-ku, Nagoya, 466-8550 Japan; 3 Regenerative & Cellular Medicine Kobe Center, Sumitomo Dainippon Pharma Co., Ltd., Chuo, Kobe, 650-0047 Japan; 4 Division of Diabetes and Endocrinology, Kobe University Hospital, Kobe, Japan; 5 Medical Center for Student Health, Kobe University, Kobe, Japan; 6 Division of Diabetes and Endocrinology, Department of Internal Medicine, Kobe University Graduate School of Medicine, Kobe, Japan; 7 Department of Diabetes and Endocrinology, Nara Medical University, Nara, 840 Shijou-Cho, Kashihara, Nara 634-8521 Japan; 8 RIKEN Center for Developmental Biology (CDB), Chuo, Kobe, 650-0047 Japan; 9 Department of iPS Cell Applied Medicine, Graduate School of Medicine, Kansai Medical University, 2-5-1 Shinmachi, Hirakata, Osaka 573-1010 Japan; 10 Center for iPS Cell Research and Application (CiRA), Kyoto University, Sakyo, Kyoto 606-8507 Japan; 11 iPSC-Based Drug Discovery and Development Team, RIKEN BioResource Research Center (BRC), Soraku, Kyoto 619-0237 Japan; 12 Medical-Risk Avoidance Based on iPS Cells Team, RIKEN Center for Advanced Intelligence Project (AIP), Tokyo, Japan

**Keywords:** Diabetes insipidus, Induced pluripotent stem cells

## Abstract

Familial neurohypophyseal diabetes insipidus (FNDI) is a degenerative disease of vasopressin (AVP) neurons. Studies in mouse in vivo models indicate that accumulation of mutant AVP prehormone is associated with FNDI pathology. However, studying human FNDI pathology in vivo is technically challenging. Therefore, an in vitro human model needs to be developed. When exogenous signals are minimized in the early phase of differentiation in vitro, mouse embryonic stem cells (ESCs)/induced pluripotent stem cells (iPSCs) differentiate into AVP neurons, whereas human ESCs/iPSCs die. Human ESCs/iPSCs are generally more similar to mouse epiblast stem cells (mEpiSCs) compared to mouse ESCs. In this study, we converted human FNDI-specific iPSCs by the naive conversion kit. Although the conversion was partial, we found improved cell survival under minimal exogenous signals and differentiation into rostral hypothalamic organoids. Overall, this method provides a simple and straightforward differentiation direction, which may improve the efficiency of hypothalamic differentiation.

## Introduction

The hypothalamus plays an essential role in maintaining physiological homeostasis. One of these is the generation and secretion of arginine vasopressin (AVP), which controls water balance. Familial neurohypophyseal diabetes insipidus (FNDI) is characterized by a progressive decrease in AVP secretion caused by the degeneration of neurons in the hypothalamus. Mutations in the AVP gene locus have been identified in patients with FNDI, and the majority of these mutations are located in the region encoding the AVP carrier protein, neurophysin II (NPII)^1^. Therefore, we generated FNDI mice with a Cys98stop (previously called Cys67stop) mutation^2^, which occurs in patients with FNDI^3^, for pathological analysis. Our previous studies using Cys98stop-knock-in FNDI mouse models^4–13^, indicated that endoplasmic reticulum (ER) stress caused by the accumulation of mutant proteins in the ER could be associated with FNDI pathology. However, further studies, including the use of human models, are needed to investigate the overall pathological mechanisms of FNDI. FNDI-related studies in humans are restricted by technical limitations because hypothalamic biopsies cannot be performed in living humans and the loss of AVP neurons in human patients with FNDI has been reported only in autopsy studies^14–16^. Therefore, AVP neurons derived from human FNDI-specific induced pluripotent stem cells (iPSCs) are a promising human model for pathological analysis and drug development.

Pluripotent stem cells can differentiate into various cells or tissues in three-dimensional cultures termed “serum-free culture of embryoid body-like aggregates with quick re-aggregation” (SFEBq)^17–28^. We previously reported the differentiation of hypothalamic neurons from mouse induced pluripotent stem cells (miPSCs) by SFEBq^29^. In these methods, the differentiation of pluripotent stem cells into neural tissue is achieved by regulating the positional information added to the medium. The hypothalamus in the embryo originates from the most rostral part of the neural plate (Fig. S1A). To induce hypothalamic differentiation of mouse iPSCs, it is important to remove exogenous signals strictly in the early stages of differentiation, and to control the positional information so that it represents the most rostral side^28,29^. The medium with minimized exogenous signals is called a growth factor-free chemically defined medium (gfCDM)^28^. With gfCDM, mouse iPSCs differentiated into the rostral hypothalamus, including AVP neurons.

The aim of this study is to induce the differentiation from human FNDI-disease specific iPSCs into AVP neurons with minimal exogenous signals, as previously described with miPSCs. In order to apply minimal exogenous signals to human iPSCs, we focused on the difference in the initial status between human iPSCs and mouse iPSCs.

Mouse epiblast stem cells (mEpiSCs) derived from post-implantation embryos^30,31^ have characteristics different from those of conventional mESCs derived from pre-implantation embryos. Nichols et al. advocated the concept of ‘naive’ and ‘primed’, which correspond to mESCs and mEpiSCs, respectively^32^. While mouse iPSCs are considered to be in a naive state, human iPSCs are recognized to be in a primed state, similar to mEpiSCs. In fact, hiPSCs and mEpiSCs share the same colony morphology and vulnerability to isolation. Therefore, in this study, we examined the hypothesis that if hiPSCs were converted to a naive state, they could be differentiated into rostral hypothalamus with minimal exogenous signals as miPSCs.

## Results

### Primed FNDI-specific hiPSCs fail to aggregate and differentiate poorly using conventional methods

Differentiation of mESCs and hESCs into hypothalamic neurons was achieved by SFEBq^28,33^, but there were differences between the methods. With minimal exogenous signals, mESCs differentiated into hypothalamic neurons whereas hESCs failed to form aggregates. As previously reported, addition of KSR, BMP4 and SAG caused hESCs to differentiate into hypothalamic precursors, but these complicated steps of differentiation did not follow the embryonic development^28,33^. Therefore, we speculated that differentiation into AVP neurons should be based on the concept of minimizing the effects of exogenous signals. First, we used FNDI iPSCs (FDI-02) to investigate which differentiation protocols were appropriate because our aim in this study was to differentiate AVP neurons from FNDI hiPSCs and identify mutant proteins. We confirmed that FDI-02 differentiated into FOXG1-positive telencephalic progenitor cells with continuous addition of 5% KSR (Fig. S2), as reported for hESCs^33^. To minimize the effects of exogenous signals, we next tried to reduce KSR concentration (0.7%, 1.0%, 1.5%, 2.5%) or shorten its incubation period (day 0–3) (Fig. S2). When the concentration of KSR at day 0 was reduced (less than 2.5%) or KSR-free gfCDM was used after day 3, the aggregates collapsed or formed cysts (Fig. S2).

Our previous study using miPSCs showed that suspending miPSC colonies to approximately 10–20 cells, rather than isolating them to a single cell for SFEBq, can facilitate miPSC aggregation^29^. Accordingly, SFEBq using clumps was performed to prevent collapse and cyst formation. When the KSR concentration at day 0 was 2.5% and KSR-free gfCDM was used after day 3, the clumps quickly aggregated. As this aggregate grew, it partially collapsed but kept partially aggregated. Even under these conditions, FNDI-iPSCs (FDI-02) differentiated into FOXG1-positive telencephalic progenitors (Fig. S2). When a lower concentration of KSR (0.7%, 1.0%, 1.5%) was used from day 0, the aggregates collapsed or formed cysts. Although SFEBq using clumps could ease the effects of reduced KSR concentration to some extent, it was difficult for primed FNDI-iPSCs (FDI-02) to differentiate into hypothalamic neurons with minimal exogenous signals.

### Partial conversion from primed to naive state improves the differentiation of FDI-02 into hypothalamic precursors

To minimize the exogenous signals needed for FNDI-iPSCs (FDI-02) differentiation, we assessed a method that enables human pluripotent stem cells to be treated in the same manner as mouse pluripotent stem cells.

Compared with conventional mESCs, human pluripotent stem cells show some distinctive features, including differences in developmental identity. In particular, mEpiSC derivation^30,31^ shows alternative pluripotency, which is associated with primitive streak-stage late epiblasts^34^. The terms ‘naive’ and ‘primed,’ which correspond to mESCs and mEpiSCs, respectively, refer to the early and late stages of epiblast ontogeny^32^. Human pluripotent stem cells share defining features with primed mEpiSCs rather than with naive mESCs. We hypothesized that these methods could help hiPSCs differentiate into hypothalamic neurons by minimizing exogenous signals.

**Figure 1:**
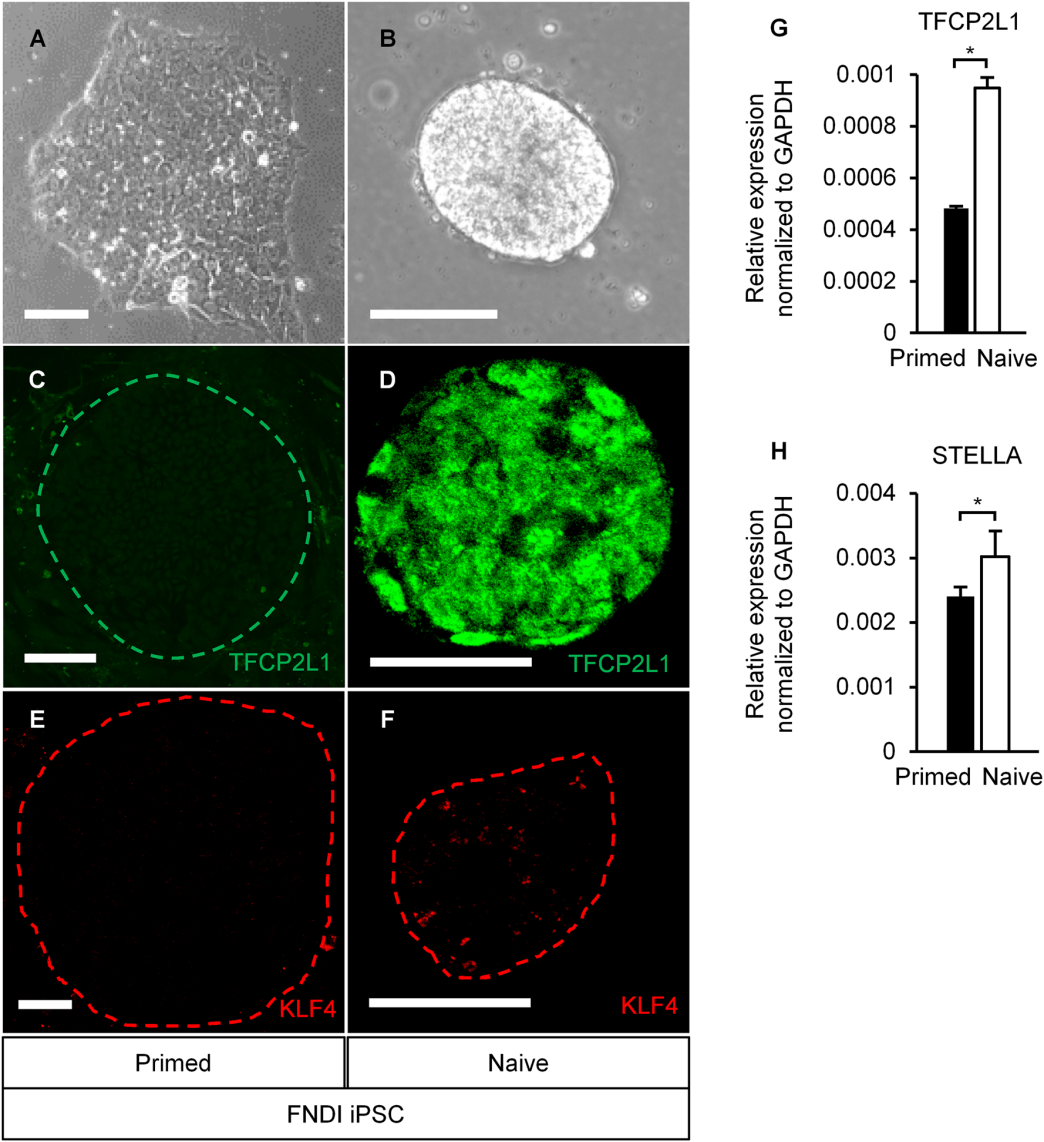
Conversion of primed hiPSCs by the naive conversion kit. (**A**) Primed hiPSC colonies had a flat morphology. Conversion by the naive conversion kit changed the morphology of colonies to tightly packed and domed (**B**), and TFCP2L1 (green) (**C,D**) and TLF4 (red) (**E,F**), naive markers, were expressed on P9. (**G,H**) Mean ± SEM. *P < 0.05, two-tailed unpaired *t*-test. The naive marker’s difference before and after conversion, TFCP2L1 and STELLA, was confirmed by RT-qPCR on P3, 4, and 5. FNDI-disease specific human iPSCs (FDI-02). Scale bars, (**A,B**) 100 µm, (**C–F**) 50 µm.

In this study, we employed RSeT™ feeder-free medium (ST-05975; STEMCELL Technologies, Vancouver, Canada), a kit for primed to naive conversion based on the articles by Chan et al., Gafni et al., Takashima et al. and Theunissen et al.^35–38^. Primed FNDI-iPSCs (FDI-02) cells were cultured under feeder-free conditions using mTeSR1™ culture medium. Thereafter, the medium was replaced with RSeT™. The colony morphology changed from flat to tightly packed and domed. We conducted immunohistochemistry on passage(P)4 and 9. TFCP2L1 and KLF4, naive markers^39^, were expressed (Fig. 1A–F). We also confirmed the significant increase of naive markers expression (TFCP2L1 and STELLA) by reverse transcription-quantitative polymerase chain reaction (RT-qPCR) (Fig. 1G,H) on P3, 4, and 5. These results demonstrated multiple features of the naive state. However, these features are less obvious than in previous reports^35–38^ and the KLF4 expression was not localized in the nucleus. Therefore, the conversion by this kit might be partial.

**Figure 2:**
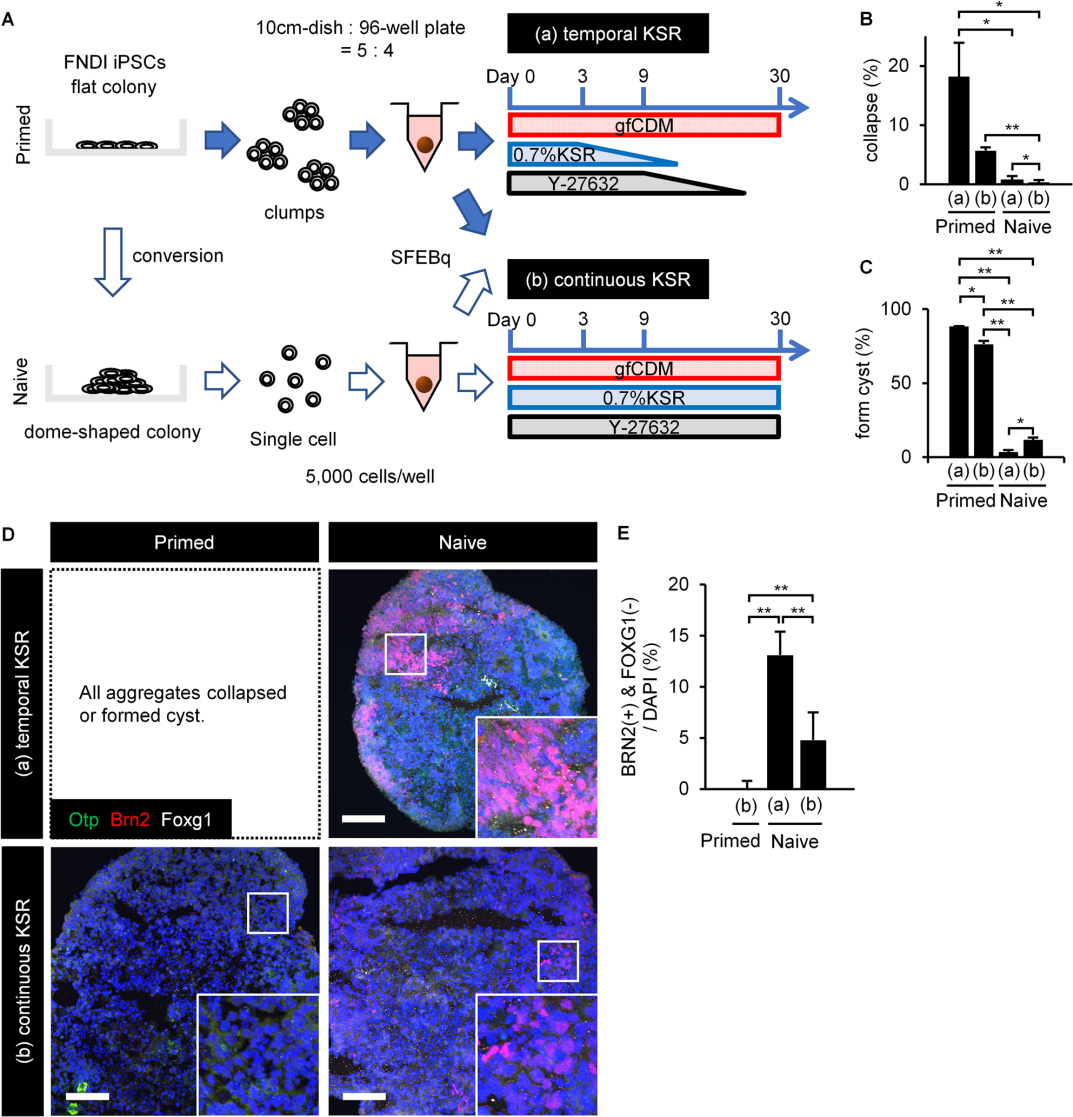
The partial conversion to the naive state showed improved aggregation on SFEBq and differentiation into hypothalamic-like cells. (**A**) Scheme of the culture protocol. Using a medium containing neither KSR nor Y-27632 from an early stage or a medium containing KSR and Y-27632 throughout, single naive hiPSCs (white arrows) were compared with primed hiPSC clumps (blue arrows). The (a) “temporal KSR” and (b) “continuous KSR” conditions were examined in both primed and naive cells. Under condition (a), KSR and Y-27632 were diluted stepwise by half medium change every 3 days with KSR(−)/Y-27632(+) medium after day 3 and KSR(−)/Y-27632(−) medium after day 9. (**B,C**) On day 30, naive aggregates showed significantly fewer changes, such as cyst formation or collapse (n = 3; three 96-well plates, that is, 288 aggregates are analyzed). Mean ± SEM. *P < 0.05; **P < 0.01, two-tailed unpaired *t*-test. On day 30, primed aggregates scarcely expressed BRN2; however, BRN2 expression was observed by conversion to the naive state (**D**), and was significantly increased by shortening the period of KSR and Y-27632 treatment (n = 8) (**E**). Mean ± SEM. *P < 0.05, **P < 0.01, two-tailed unpaired *t*-test. OTP (green), BRN2 (red), FOXG1 (white). The increased expression of the AVP precursor marker, BRN2, indicated that minimization of exogenous signals improved the efficiency of differentiation into hypothalamic-like cells. For all relevant panels, nuclear counterstaining was performed with DAPI (blue). Scale bars, 100 µm.

SFEBq was performed with partially naive FNDI-iPS (FDI-02) single cells and primed FDI-02 clumps. The use of a medium containing neither KSR nor Y-27632, a Rho-associated kinase (ROCK) inhibitor, from an early stage was compared with a medium containing both throughout the culture (Fig. 2A). Even when the initial concentration of KSR was reduced to 0.7%, the single cells and clumps quickly aggregated under all conditions. Most primed aggregates formed cysts or collapsed over time, whereas partially naive aggregates maintained a healthy state. The partially naive aggregates showed significantly fewer changes on day 30 (Fig. 2B,C).

Aggregates were evaluated by immunohistochemistry. The expression of BRN2, an AVP precursor marker^40,41^, was significantly increased in partially naive aggregates in the shortened period condition with KSR and Y-27632 (Fig. 2D,E). We demonstrated that not only the conversion by the naive conversion kit but also the minimization of exogenous signals was essential for the differentiation into AVP neurons. We also confirmed that the partially naive aggregates contained very few FOXG1 positive cells. This is consistent with increased hypothalamic differentiation with minimal exogenous signals.

In summary, conversion by the naive conversion kit enabled minimization of exogenous signals in SFEBq with hiPSCs. Furthermore, the minimization of exogenous signals improved the efficiency of differentiation into hypothalamic-like cells.

### Hypothalamic organoids derived from FDI-02 expressed mutant NPII

**Figure 3:**
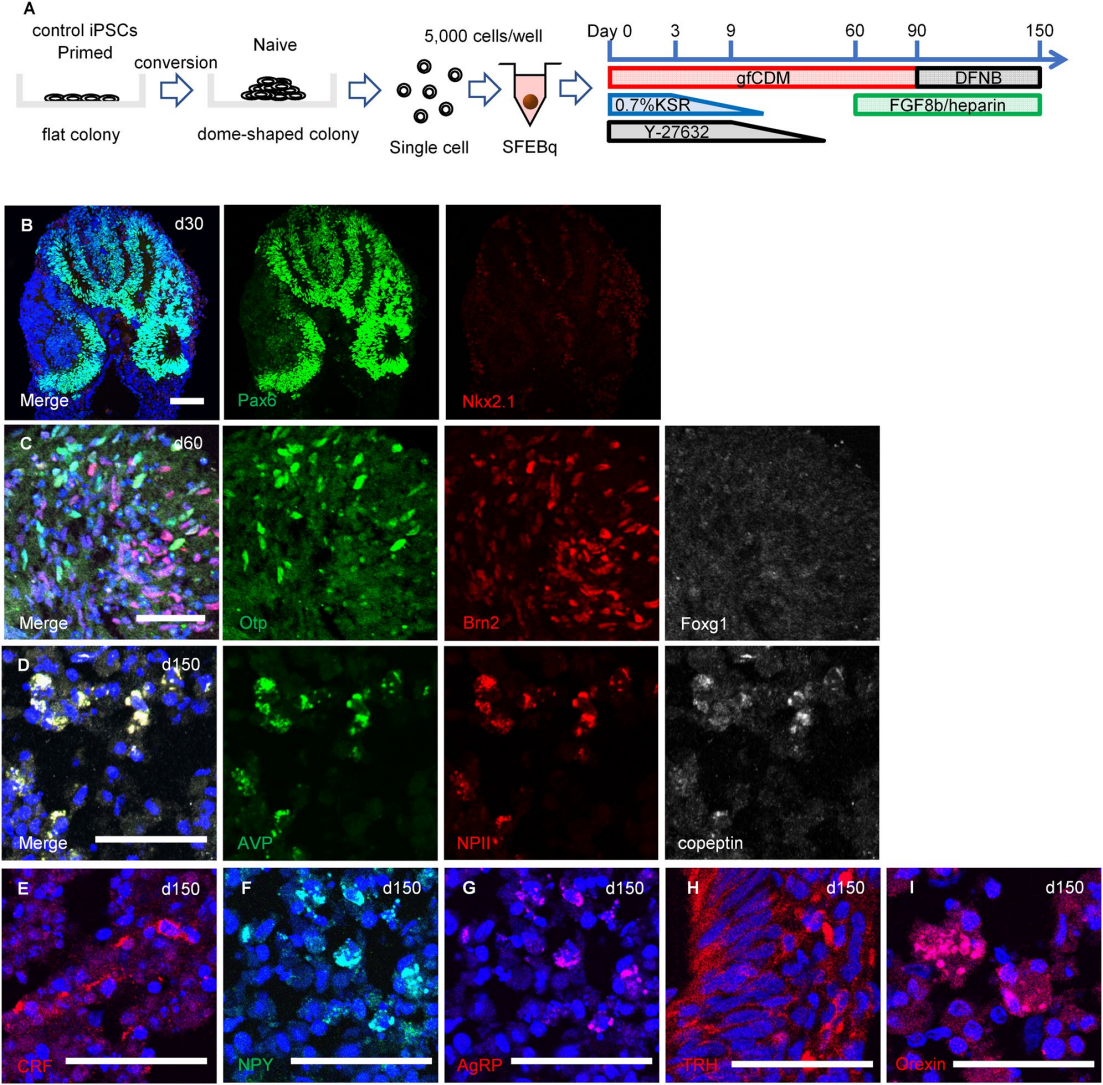
The partial conversion to the naive state enabled hiPSCs to differentiate into AVP neurons and hypothalamic organoid. (**A**) Scheme of the culture protocol. SFEBq using 201B7 cells recapitulated embryogenesis; on day 30, dorsal hypothalamic progenitor cells [PAX6+ (green), NKX2.1− (red)] (**B**), and on day 60, AVP precursor cells [OTP+ (green), BRN2+ (red)] (**C**), were sequentially identified. FOXG1 (white). (**D**) On day 150, differentiation into AVP neurons was achieved, with co-expression of pro-AVP components: AVP (green), NPII (red), and copeptin (white). Day 150 aggregates also expressed other hypothalamic markers such as CRF (**E**), NPY (**F**), AgRP (**G**), TRH (**H**), and Orexin (**I**). It is demonstrated that these cells are not only AVP neurons but also hypothalamic organoids. For all relevant panels, nuclear counterstaining was performed with DAPI (blue). Scale bars, (**B**) 100 µm, (**C–I**) 50 µm.

We have previously reported that the addition of FGF8b to late-stage culture improves the efficiency of AVP neuron differentiation^29^. Therefore, we adopted this method and established an overall protocol (Fig. 3A).

**Figure 4:**
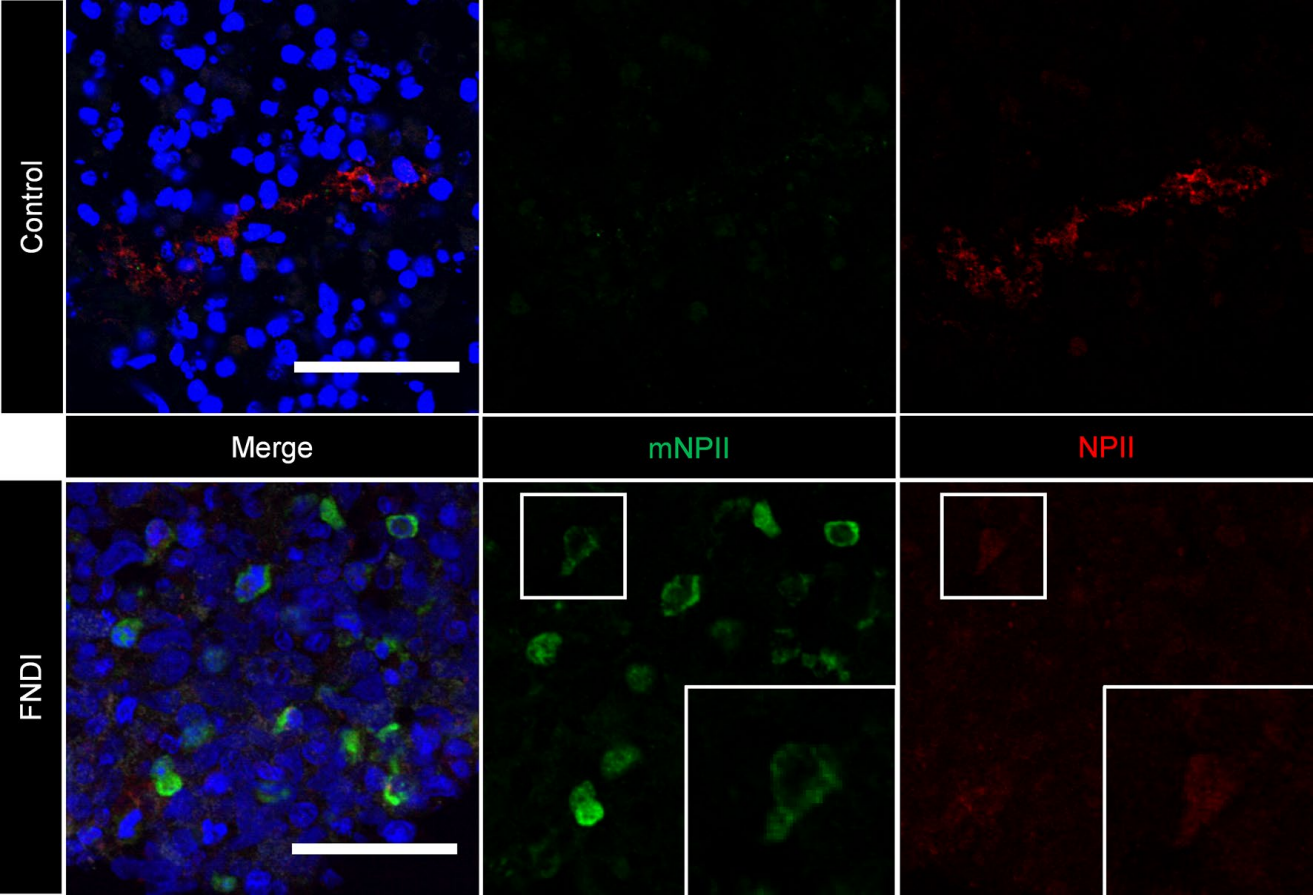
FNDI-specific hiPSCs generated mutant NPII. Expression of normal NPII (red) was observed in SFEBq from FDI-02. Additionally, the expression of mutant NPII (mNPII) (green) was confirmed, which was not expressed in 201B7-derived AVP neurons. For all relevant panels, nuclear counterstaining was performed with DAPI (blue). Scale bars, 50 µm.

Human iPSC cell line, 201B7 was converted as FDI-02 and SFEBq was performed according to the established protocol as a control. Dorsal hypothalamic progenitor (PAX6+, NKX2.1−) and AVP precursor (OTP+, BRN2+) cells were sequentially identified (Fig. 3B,C). On day 150, we observed cells co-expressing AVP, NPII, and copeptin, which are components of pro-AVP, and thus achieved differentiation into AVP neurons (Fig. 3D). Using the same method, FDI-02 differentiated into AVP neurons. Simultaneously, we confirmed the expression of other hypothalamic markers such as CRF, NPY, AgRP, TRH, and Orexin (Fig. 3E–I), which demonstrated successful differentiation to AVP neurons as well as hypothalamic organoids. In FNDI iPSCs, we also confirmed the sequential differentiation into hypothalamic organoids (Fig. S3). Finally, we proved the expression of mutant NPII, which was not expressed in 201B7-derived AVP neurons (Fig. 4).

## Discussion

In this study, we investigated a novel perspective in the differentiation of AVP neurons, regarding whether cells were in a naive or primed state before differentiation, rather than considering the early or later stages of the differentiation protocol^28,29,33^. We found two advantages. First, the trial of the conversion of cells from primed to naive state solved problems such as decreased survival, collapse of aggregates, and cyst formation by minimizing exogenous signals in the SFEBq method for the differentiation of human iPSCs into AVP neurons. Second, even after solving these problems, the strict removal of exogenous signals further increased the rate of cells positive for BRN2, which is an essential transcription factor for AVP differentiation^40–44^. These findings indicate an improved efficiency of AVP neuron differentiation.

The differentiation potential of pluripotent stem cells varies among cell lines^45^. When differentiating the rostral part of the central nervous system such as the hypothalamus, the use of naive cells could be one way to solve this problem. It is difficult to differentiate the primed hESCs/hiPSCs into rostral hypothalamic neurons because they cannot survive in culture conditions with minimal exogenous signals. Historically, it is known that dissociation of primed hESCs causes apoptosis due to the Rho-high/Rac-low state^46^. Thus, treatment with ROCK inhibitors is very effective for the dissociation process of primed hESCs^47^. Using a ROCK inhibitor, primed hESCs/hiPSCs can be differentiated into non-rostral nervous systems such as the telencephalon^17,18^, cerebellum^22^, and spinal cord^24^. However, for inducing the rostral part of the central nervous system, such as AVP neurons, additional conditions are required to induce the rostral edge of the neural plate^28,29^. In the primed hESC/hiPSC cultures, use of the ROCK inhibitor alone cannot maintain their aggregation in the state of minimal exogenous signals. Nutrients such as KSR must be added to some extent for their survival, resulting in their differentiation into FOXG1-positive cerebral tissues. Additional BMP4 and SHH signals are necessary to primed hESCs for repositioning from the cerebrum to the hypothalamus^33^. This means that a two-step process takes place in which the hESCs, once posteriorised, are repositioned anteriorly (Fig. S1B). In this report, the conversion of hiPSCs by the naive conversion kit, which minimized the addition of ROCK inhibitors and KSRs, may have resulted in a simpler and more straightforward direction of differentiation and improved the efficiency of differentiation to the rostral hypothalamus.

The stability of the naive conversion is a significant issue. The kit manufacturer recommends monitoring their karyotype after 10 passages. Therefore, we used iPSCs within 10 passages after conversion and confirmed their expression of naive markers at P9 by immunohistochemistry. The converted hiPSCs in this study kept their feature when we conducted SFEBq.

FNDI is a hereditary neurodegenerative intractable disease for which there is currently no fundamental treatment. Analyses of our FNDI model mice, which possess the Cys98stop mutation^2,3^, revealed that mutant NPII was accumulated in the ER-associated compartment (ERAC) in AVP neurons^4,5^. In this study, we clearly demonstrated mutant NPII expression in FNDI-disease specific hiPSC-derived AVP neurons. Accumulation of mutant proteins in the ER is implicated in the pathophysiology of many diseases, including FNDI^1,6–13,48,49^. Furthermore, novel ER stress-reducing drugs have been reported^50^. Therefore, FNDI-disease specific hiPSC-derived AVP neurons are a promising human model of ER stress and are a valuable resource for drug development. We plan to analyze characteristic structures, such as the ERAC, which was confirmed in mice by electron microscopy, in FNDI-specific hiPSC-derived AVP neurons. We would also like to undertake a functional investigation for pathological analysis and drug development in future studies.

There is a limitation about the naive state in this study. The conversion was performed with RSeT™ feeder-free medium^35–38^. Although we were able to identify multiple features of the naive state, the conversion to the naive state by this kit might be partial as we described in Results. Even this partial conversion was enough to play an essential role in hypothalamic differentiation. We wonder if the ‘partial’ conversion may have been more effective for hypothalamic differentiation. There are several other reports concerning conversion to the naive type^51–56^. Further studies are needed to determine which way of naive conversion for hiPSC is the best in differentiating human rostral hypothalamus. In addition, the positive areas of hypothalamic markers, including AVP, were localized to a small part of the aggregates. Based on this study, we will continue to improve the differentiation method, analyze the expression pattern of mutant proteins in detail, and research drug reactivity.

## Methods

These experiments were approved by the ethics committee of Nagoya University and performed in accordance with relevant guidelines and regulations. Informed consent was obtained from all subjects.

### Generation of FNDI hiPSCs

We generated iPSCs from a patient with FNDI with a Cys98Stop mutation^3^ based on ethical approval from Nagoya University Committee (2013-0228-2). To establish patient-derived iPSCs, peripheral blood monocular cells of the patient were reprogrammed using episomal vectors (expressing OCT3/4, SOX2, KLF4, L-MYC, LIN28, EBNA1, and p53shRNA) as described previously^57,58^. The iPSCs showed embryonic stem cell-like morphology and normal karyotype (Fig. S4A). These cells expressed the undifferentiated markers (Fig. S4B,C) and could differentiate into the three germ layers in vitro (Fig. S4D), indicating that these cells were pluripotent (deposited in RIKEN BRC as HPS1011 and HPS1904). In this study, we used a cell line named FDI-02, which corresponds to HPS1904. As a control, we used the 201B7 cell line [Research Resource Identifier (RRID): CVCL_A324].

### Differentiation into three germ layers

To generate embryoid bodies (EBs), iPSCs harvested with TrypLE Select (A1285901; Gibco, Waltham, MA, USA) were cultured in EB medium (10565018; DMEM/F12/Glutamax (Gibco), 20% KSR (Gibco), 1× nonessential amino acids (11140-050; Gibco), penicillin/streptomycin (15140-122; Gibco) and 10 μM Y-27632 (Nacalai Tesque, Kyoto, Japan)) on a low-attachment surface V-bottom 96-well plates (Sumitomo Bakelite, Tokyo, Japan). On day 11, EBs were collected and seeded into Matrigel-coated 24-well plates, resulting in differentiation into three germ layers in 7 days.

### Thawing and maintenance of primed hiPSCs

We prepared mouse embryonic fibroblasts (MEFs) (KBL9284600; Kitayama Labes, Nagano, Japan) inactivated by mitomycin C treatment on 0.1% gelatin-coated dishes (1.2 × 10^6^ cells/10 cm dish). Mice were not directly involved in the study. Primed hiPSCs frozen in liquid nitrogen were thawed as quickly as possible in warmed maintenance medium, comprising DMEM/F12 (D6421; Sigma, St. Louis, Missouri, USA) supplemented with 20% (v/v) KSR (lot No. 1517496; Invitrogen, Waltham, Massachusetts, USA), 0.1 mM non-essential amino acids (11140-050; Gibco), 2 mM l-glutamine (25030-081; Gibco), 5 ng/mL recombinant human basic FGF (068-04544; Wako, Osaka, Japan), and 0.1 mM 2-mercaptoethanol (131-14572; Wako. Cells suspended in maintenance medium were distributed on MEF-coated dishes and maintained in a CO_2_ incubator under 2% CO_2_ at 37 °C. The medium was changed daily.

For passaging, primed hiPSC colonies were harvested by incubation in 0.25% (w/v) trypsin and 0.1 mg/mL collagenase IV in PBS containing 20% (v/v) KSR and 1 mM CaCl_2_ for 6–8 min at 37 °C. The harvested clumps were broken into smaller pieces by gentle pipetting. The passages were performed at a split ratio of 1:4–6.

### Conversion of hiPSCs by RSeT™ and maintenance in culture

hiPSCs harvested during passaging, were suspended in mTeSR1™ medium (ST-85850; STEMCELL Technologies), distributed on a Matrigel-coated dish (354277; Corning, Corning, New York, USA), and incubated under 5% CO_2_ at 37 °C. After 24–36 h, the mTeSR1 medium was replaced with RSeT™ feeder-free medium (ST-05975; STEMCELL Technologies) and cells were incubated under hypoxic conditions (5% O_2_, 5% CO_2_) at 37 °C. The medium was changed every second day. Reset to the naive state was confirmed by changes in colony morphology and immunohistochemistry.

For passaging, naive hiPSCs were harvested using TrypLE Express (12605-010; Thermo Fisher, Waltham, Massachusetts, USA). Cells were counted and 1.1 × 10^6^ cells were distributed on a 10 cm Matrigel-coated dish. The medium was changed after 24–36 h and thereafter, every second day. This passaging procedure was performed every 4–6 days. These cells were used within 10 passages for the differentiation experiments.

### RT-qPCR

We isolated total RNA with the RNeasy kit (74136; QIAGEN, Hilden, Germany) and made complementary DNA (cDNA) from 5 µg of total RNA with SuperScript III (12574026; Invitrogen) and oligo-dT primers. We performed qPCR with TaqMan Fast Universal Master Mix (4352042; Applied Biosystems, Waltham, Massachusetts, USA) and TaqMan probes (Applied Biosystems) or the Universal Probe Library (UPL, Roche) system. We conducted three technical replicates. We applied GAPDH (4352934T; Applied Biosystems) as an endogenous control to normalize expression. The information of primers and probes are as below; TFCP2L1 (TaqMan probe: Hs00232708_m1), KLF4 (TaqMan probe: Hs00358836_m1), and STELLA (UPL: #80, primer: U_STELLA R tggtagcaatttgaggctctg, U_STELLA L atcggcgtcttgacacaac).

### Differentiation of hiPSCs into hypothalamic-like neurons

Hypothalamic differentiation was performed using the SFEBq culture. Primed hiPSCs were harvested and dissociated into single cells using TrypLE Express (12605-010; Invitrogen) containing 0.05 mg/mL DNase I (11284932001; Roche, Basel, Switzerland) and 10 mM Y-27632 (034-24024; Wako). Cells were then suspended in gfCDM differentiation medium [1:1 Iscove’s-modified Dulbecco’s medium (IMDM), GlutaMAX™ supplement (31980-030; Gibco)/Ham’s F-12 nutrient mix, GlutaMAX™ supplement (31765-035; Gibco), 250 mg/mL BSA (A3156; Sigma), 1× chemically defined lipid concentrate (11905-031; Gibco), and 438 μM 1-thioglycerol (M6145; Sigma)] and distributed in low-cell-adhesion 96-well plates with V-bottomed conical wells (MS-9096V; Sumitomo Bakelite, Tokyo, Japan) at 5,000 cells in 100 µL/well. When the SFEBq culture was started, gfCDM contained 0.7–5.0% KSR and 20 mM Y-27632, which were diluted in a step-wise manner by replacing one-half of the medium with new medium without KSR or Y-27632. In another method, harvested primed hiPSCs were broken by gentle pipetting. Eighty percent of the broken clumps from one confluent 10 cm dish were suspended in gfCDM and distributed into one 96-well plate. Naive hiPSCs were harvested using TrypLE Express and dissociated into single cells without mechanical disruption. These were then suspended in gfCDM (5000 cells in 100 µL/well) and distributed in 96-well plates. All SFEBq cultures were incubated in 5% CO_2_ at 37 °C. The concentrations of KSR and Y-27632 in the medium used for the medium change were adjusted for each experiment.

We designated the day when the culture was started as day 0. On day 3, 100 μL gfCDM was added to each well. From day 6, half of the medium was changed every 3 days. In experiments to minimize the KSR addition, the concentration of KSR at day 0 was reduced or KSR-free gfCDM was used after day 3. After culturing in a 96-well plate for 30 days, the aggregates were transferred to a 10 cm dish for suspension culture. From day 60, 100 ng/mL FGF8b (423-F8; R&D Systems, Minneapolis, MN, USA) and 5 μg/mL heparin (NIPRO, Osaka, Japan) were added to the differentiation medium. From day 90, the medium was completely replaced with DMEM/F12 supplemented with glucose, N2, and B27 [DFNB medium: DMEM/F12 (D8900; Sigma) supplemented with 3.85 g/L glucose (07–0680-5; Sigma), 1.2 g/L sodium hydrogen carbonate (28–1850-5; Sigma), penicillin/streptomycin (50 U/mL and 50 μg/mL, respectively) (15140-122; Gibco), N2 (175020-01; Gibco), B27 (125870-01; Gibco), and 10 ng/mL CNTF (257-NT; R&D Systems)] with 100 ng/mL FGF8b and 5 μg/mL heparin.

### Immunohistochemistry

Aggregates were fixed with 4% PFA for 10–15 min and embedded in OCT compound (4583; Sakura Finetek, Tokyo, Japan). Ten micron thick sections were cut using a cryostat, mounted on slides, and fixed in 4% PFA for 10 min.

Sections were permeabilized using 0.3% Triton X-100/PBS and washed with PBS. Subsequently, they were incubated in 2% (w/v) skimmed milk/PBS for 1 h at RT and then with primary antibodies diluted in 2% skimmed milk/PBS, overnight at 4 °C. The next day, they were washed with 0.05% Tween 20/PBS and reacted with 4,6-diamidino-2-phenylindole (DAPI; D523; Dojindo, Kumamoto, Japan) and secondary antibodies diluted in 2% skimmed milk/PBS for 2 h at RT. Subsequently, they were washed with 0.05% Tween 20/PBS and mounted in SlowFade™ Diamond (S36972; Thermo Fisher Scientific).

Primary antibodies were used against the following molecules (with dilutions): AVP (T5048; guinea pig; 1:2000; Peninsula; RRID:AB_2313978), BRN2 (sc-6029; goat; 1:500; Santa Cruz; RRID:AB_2167385), Copeptin (BORIS Y; rabbit; 1:1000; Woomera Therapeutics), E-cad (M108; rat; 1:50; TAKARA), FOXG1 (M227; rabbit; 1:1000; TAKARA; RRID:AB_2827749), mutant NPII [AFT965002-B(2B); rabbit; 1:1000; custom], NANOG (4903; rabbit; 1:500; Cell signaling; RRID:AB_10559205), NKX2.1 (16108; mouse; 1:100; PROGEN; RRID:AB_1543129), NPII (MABN845; mouse; 1:1000; Millipore; RRID:AB_2819363), OCT3/4 (611202; mouse; 1:100; BD biosciences; RRID:AB_398736), OTP (MS1535GS; guinea pig; 1:1000; Takara), PAX6 (ab195045; rabbit; 1:350; abcam; RRID:AB_2750924), SMA (M0851; mouse; 1:500; DAKO; RRID:AB_2223500), SOX17 (81778; rabbit; 1:3000; Cell Signaling; RRID:AB_2650582), SSEA-4 (MAB4304; mouse; 1:2000; Millipore; RRID:AB_177629), TFCP2L1 (AF5726; goat; 1:200; R&D Systems; RRID:AB_2202564), TRA1-60 (MAB4360; mouse; 1:200; Millipore; RRID:AB_2119183), and TUJ1 (MMS-435P; mouse; 1:500; Covance; RRID:AB_2313773).

### Statistics and reproducibility

We have described the exact *n* values for each experiment in the main text and figure legends. IBM SPSS Statistics (IBM, Armonk, New York, USA) was used for the statistical analyses. Two-group comparisons were performed using the two-tailed unpaired *t-*test. Significance was set at P < 0.05.

## Supplementary Information

Supplementary Figure S1.

Supplementary Figure S2.

Supplementary Figure S3.

Supplementary Figure S4.

## Data Availability

The datasets generated during and/or analyzed during this study are available from the corresponding author upon reasonable request.
