# Supplementary figures and images for: Differentiation of human induced pluripotent stem cells into hypothalamic vasopressin neurons with minimal exogenous signals and partial conversion to the naive state
Source: Sci Rep. 2022 Oct 17;12:17381. doi: 10.1038/s41598-022-22405-8 (PMC9576732; doi:10.1038/s41598-022-22405-8)

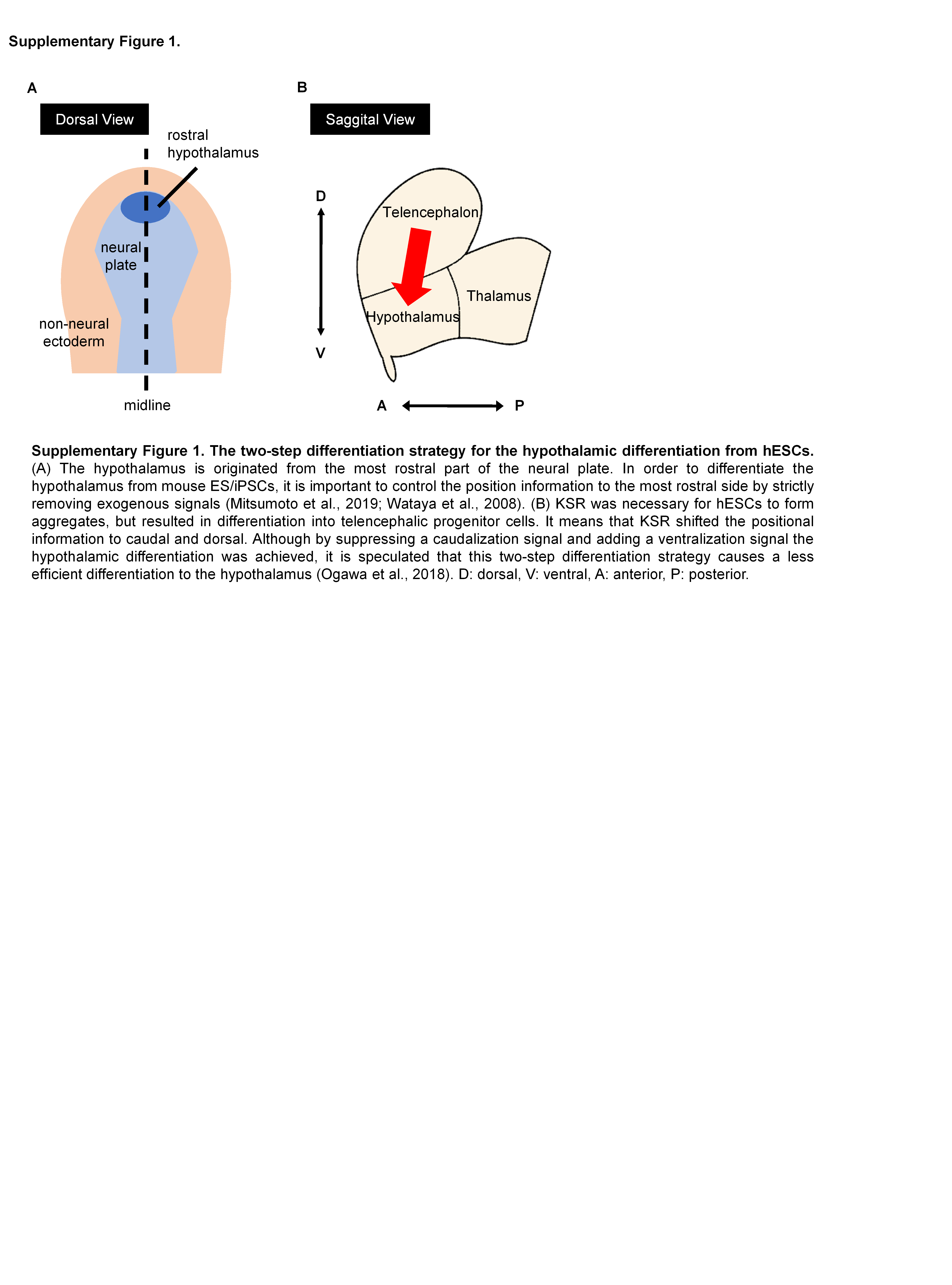

Supplement: Supplementary file 1 — Supplementary Figure S1. [file 41598_2022_22405_MOESM1_ESM.tif]

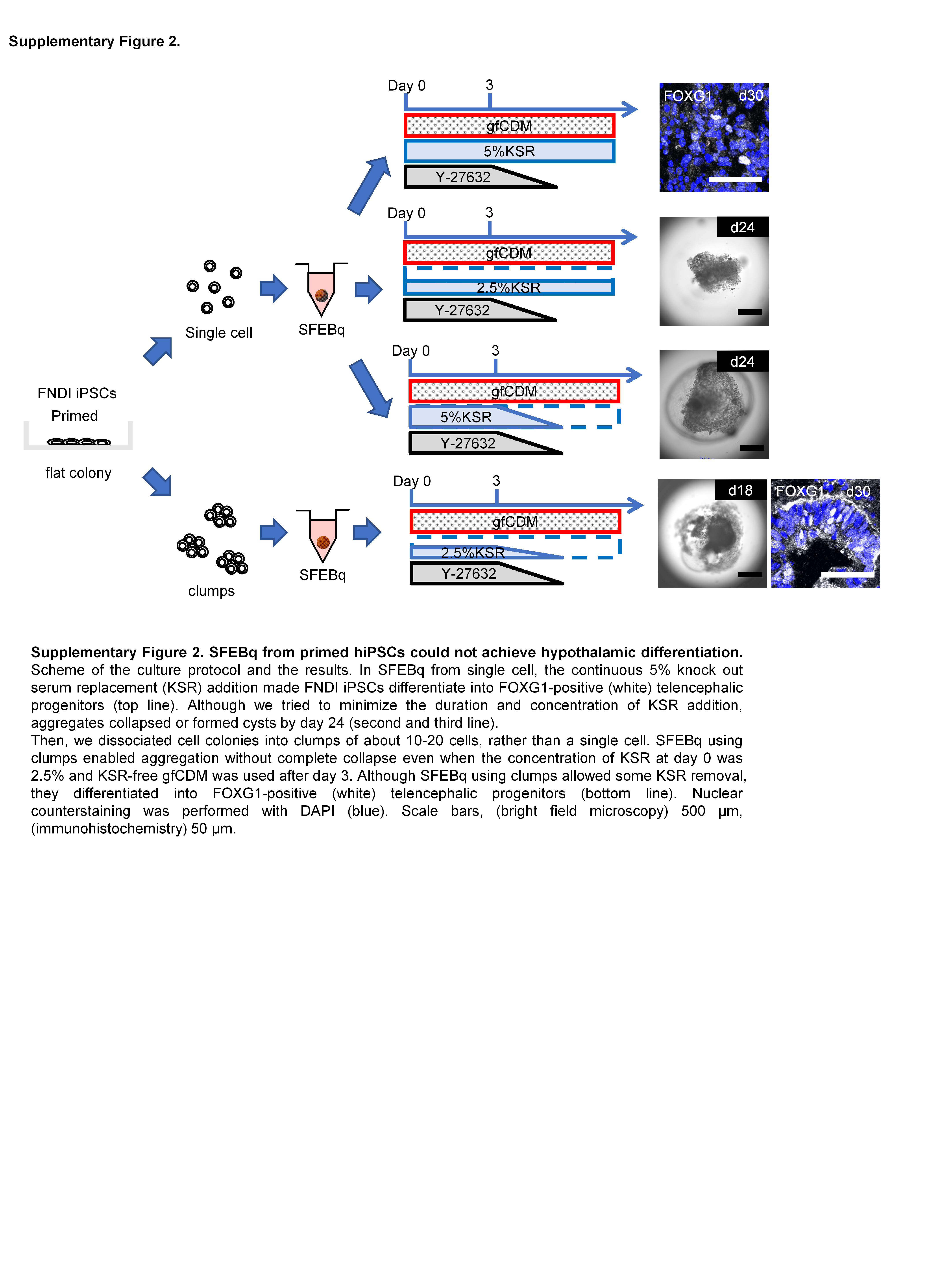

Supplement: Supplementary file 2 — Supplementary Figure S2. [file 41598_2022_22405_MOESM2_ESM.tif]

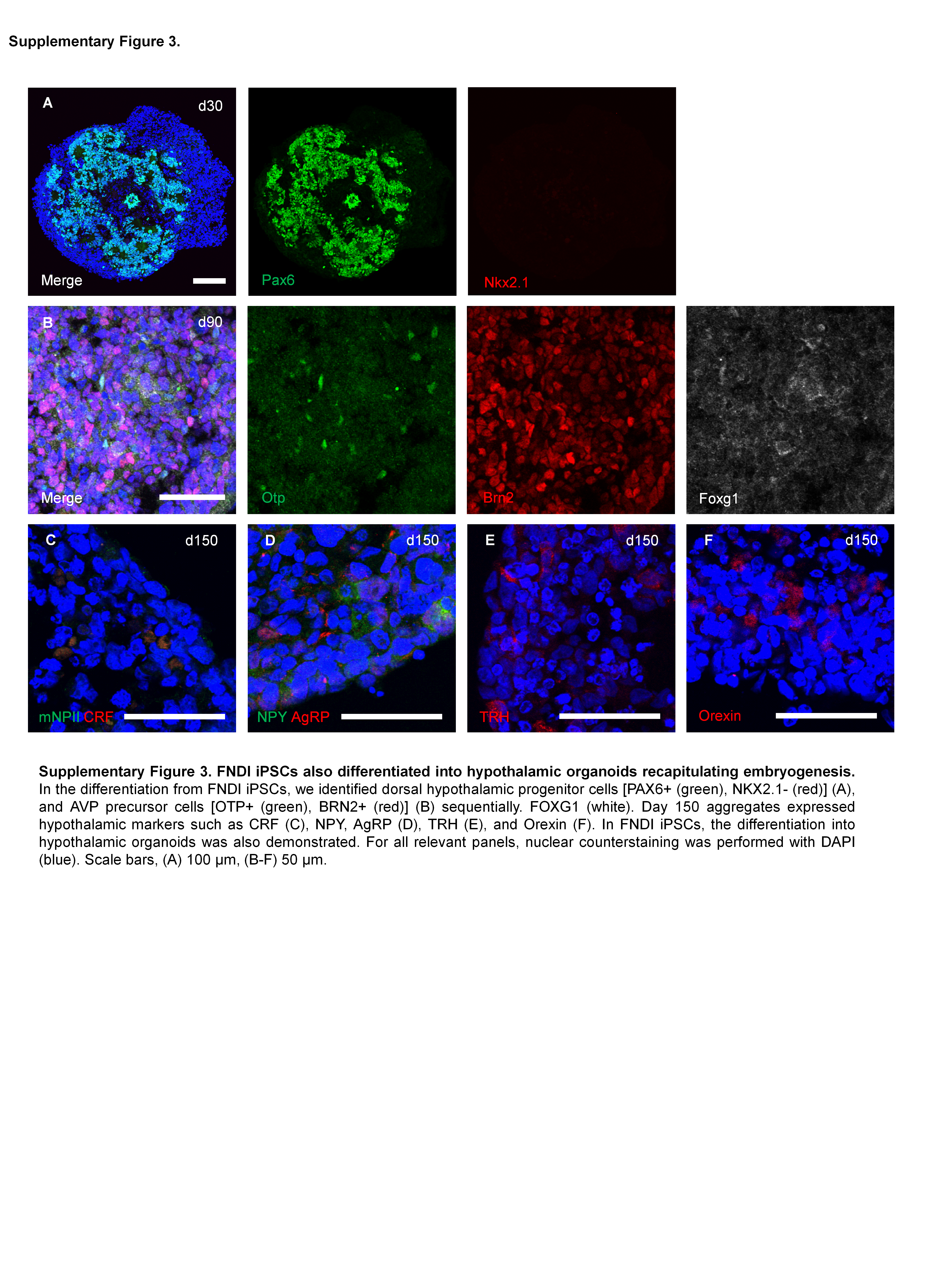

Supplement: Supplementary file 3 — Supplementary Figure S3. [file 41598_2022_22405_MOESM3_ESM.tif]

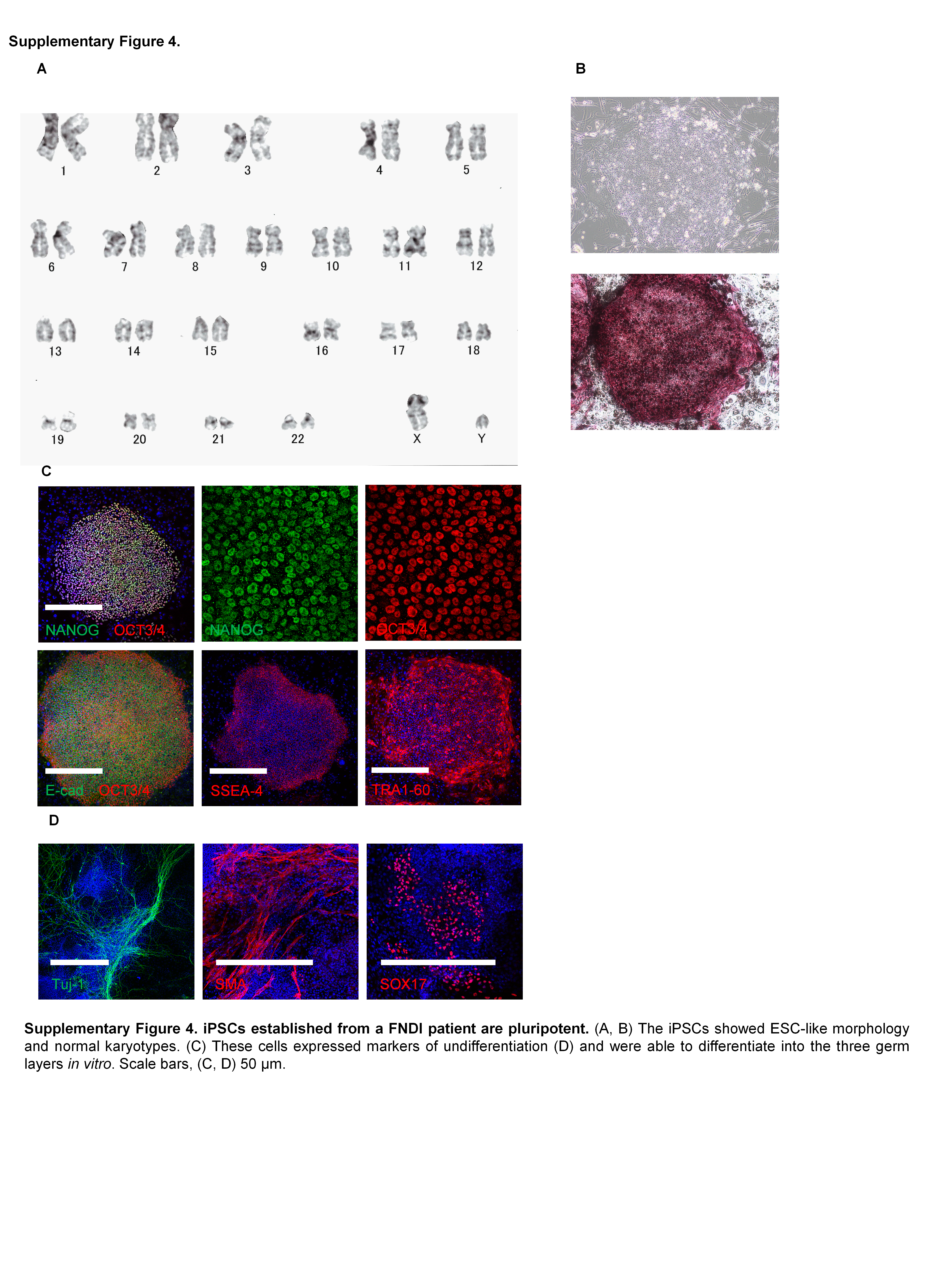

Supplement: Supplementary file 4 — Supplementary Figure S4. [file 41598_2022_22405_MOESM4_ESM.tif]
